# Supplementary material for: Multi-Mechanistic and Therapeutic Exploration of Nephroprotective Effect of Traditional Ayurvedic Polyherbal Formulation Using In Silico, In Vitro and In Vivo Approaches
Source: Biomedicines. 2023 Jan 9;11(1):168. doi: 10.3390/biomedicines11010168 (PMC9855918; doi:10.3390/biomedicines11010168)
Supplement: Supplementary file 1 [file biomedicines-11-00168-s001.zip › biomedicines-2077880-supplementary.pdf]

Table S1: Composition of NEERI KFT and their ethnopharmacological claim for kidney/urinary, urolithiatic disorder via immunomodulation and anti-inflammatory by API, UPI, Unani texts, and reports of current evidences.

Sr.No	Common name	Botanical name and source	Concentration (mg)	Traditional claim		
				API	UPI	Current Evidences
1.	Punernava	*Boerhavia diffusa* L., root	1000	Mutrala (Diuretic), Vatakantaka/sothahara (Anti-inflammation) API, Part-I, Vol-I, V & IX, 140, 211 & 103		(Mishra et al., 2014), (Singh et al., 2020)
2.	Kasni	*Cichorium intybus* L.,stem	600	-	Mudir-e-Baul (Diuretic) UPI, Part-I Vol-VI 96	(Bahmani et al., 2015)
3.	Makoya	*Solanum nigrum* L., fruit	500	Prameha (Urinary disorder/ increased frequency and turbidity of urine) API, Part-I, Vol-II, 68	Muhallil-e-auram (Anti-inflammatory) UPI, Part-I, Vol-VI, 93	(Azarkish et al., 2017)

**4.**	Giloe	*Tinospora cordifolia* (Willd.) Miers, stem	500	Prameha (Urinary disorder/ increased frequency and turbidity of urine)<br>API, Part-I, Vol-I, 41	Mudirr-e-Baul (Diuretic)<br>UPI, Part-I, Vol-I, 31	(Singh and Chaudhuri, 2017)
**5.**	Kamal fool	*Nelumbo nucifera* Gaertn., flower	400	Mutra virajaniya (Urinary depigmenter)<br>API, Part-I, Vol-II, 70		(Sharma et al., 2020)
**6.**	Palash	*Butea monosperma* (Lam.) Taub., flower	300	Mutrakrcchra (Dysuria) and Prameha (Urinary disorder/ increased frequency and turbidity of urine)<br>API, Part-I, Vol-V, 162	Mudirr-e-Baul (Diuretic)<br>UPI, Part-I Vol-II, 83	(Singh et al., 2020)
**7.**	Gokshru	*Tribulus terrestris* L., fruit	300	Mutrakrcchra (Dysuria)<br>API, Part-I, Vol-1, 40	Mudirr-e-Baul (Diuretic), Mufattit-e-Hasat (anti-lithotriptic)<br>UPI, Part-I Vol-I, 53	(Kaushik et al., 2019)

**8.**	Sirisa	*Albizia lebbeck* (L.) Benth., stem	200	-		(Ahmed et al., 2014)
**9.**	Lal chandan	*Pterocarpus santalinus* L.f., stem	200	-	Bol-ud-dam(Heamaturia) Tanqueehul Mufradat (Tanqeehul mufradat, Pg-169)	(Bulle et al., 2016)
**10.**	Haridra	*Curcuma longa* L., rhizome	200	Prameha (Urinary disorder/ increased frequency and turbidity of urine) API, Part-I, Vol-I, 61	Muhallil-e-Auram (Anti-inflammatory) Tanqueehul Mufradat (Tanqeehul mufradat, Pg-108)	(Ghosh et al., 2014)
**11.**	Ushira/Khas	*Vetiveria zizanioides* (L.) Nash, stem	150	Mutrakrcchra (Dysuria) API, Part-I, Vol-III, 221		
**12.**	Aanantmool	*Hemidesmus indicus* (L.) R. Br. ex Schult., stem	150	Raktavikara (Blood detoxification) API, Part-I, Vol-I, 107		(Sandeep and Krishnan Nair, 2010)

**13.**	Dhania	*Coriandrum sativum* L., fruit	100	Mutrala (Diuretic) API, Part-I, Vol-I, 31	Muhallil-e-waram (Anti-inflammatory) UPI, Part I, Vol-I, 57	(Lakhera et al., 2015)
**14.**	Sigru	*Moringa oleifera* Lam., seed	100	Mutrasarkara (Normalization of glucose in urine), API, Part-I, Vol-IV, 111		(Akinrinde et al., 2020)
**15.**	Varun	*Crataeva nurvala* Buch. Ham. stem	100	Raktavikara (Disorders of blood) API, Part-I, Vol-VI, 87		(Sharma et al., 2020)
**16.**	Chaulai	*Amaranthus spinosus* L. seed	100	-		(Amuthan et al., 2012)
**17.**	Revand chini	*Rheum emodi* Wall., rhizome	100	-	Mudirr-e-Baul(Diuretric) UPI, Part-I, Vol-II, 92	(Alam et al., 2005)

**18.**	Kakri Beej	*Cucumis utilissimus/melo* Roxb., seeds	100	Mutrakrcchra (Dysuria) API, Part-I, Vol-II, 39	Mudirr-e-Baul (Diuretic) UPI, Part-I, Vol-IV, 64	(Saleem et al., 2019)
**19.**	Papita Jad	*Carica papaya* L., root	50	Mutraroga (Urinery diseases), API, Part-I, Vol-VI, 89		(Naggayi et al., 2015)

References

Ahmed, D., Kumar, V., Verma, A., Gupta, P.S., Kumar, H., Dhingra, V., Mishra, V., Sharma, M., 2014. Antidiabetic, renal/hepatic/pancreas/cardiac protective and antioxidant potential of methanol/dichloromethane extract of Albizzia Lebbeck Benth. stem bark (ALEx) on streptozotocin induced diabetic rats. BMC Complement. Altern. Med. 14, 243. https://doi.org/10.1186/1472-6882-14-243

Akinrinde, A.S., Oduwole, O., Akinrinmade, F.J., Bolaji-Alabi, F.B., 2020. Nephroprotective effect of methanol extract of moringa oleifera leaves on acute kidney injury induced by ischemia-reperfusion in rats. Afr. Health Sci. 20, 1382–1396.

Alam, M.M.A., Javed, K., Jafri, M.A., 2005. Effect of Rheum emodi (Revand Hindi) on renal functions in rats. J. Ethnopharmacol. 96, 121-125.

Amuthan, A., Chogtu, B., Bairy, K.L., Sudhakar, Prakash, M., 2012. Evaluation of diuretic activity of Amaranthus spinosus Linn. aqueous extract in Wistar rats. J. Ethnopharmacol. 140, 424-427.

Azarkish, F., Hashemi, K., Talebi, A., Kamalinejad, M., Soltani, N., Pouladian, N., 2017. Effect of the administration of Solanum nigrum fruit on prevention of diabetic nephropathy in streptozotocin-induced diabetic rats. Pharmacognosy Res. https://doi.org/10.4103/pr.pr_47_17

Bahmani, M., Shahinfard, N., Rafieian-Kopaei, M., Saki, K., Shahsavari, S., Taherikalani, M., Ghafourian, S., Baharvand-Ahmadi, B., 2015. Chicory: A review on ethnobotanical effects of Cichorium intybus L. J. Chem. Pharm. Sci.ISSN:0974-2115

Bulle, S., Reddy, V.D., Hebbani, A.V., Padmavathi, P., Challa, C., Puvvada, P.K., Repalle, E., Nayakanti, D., Aluganti Narasimhulu, C., Nallanchakravarthula, V., 2016. Nephro-protective action of P. santalinus against alcohol-induced biochemical alterations and oxidative damage in rats. Biomed. Pharmacother. 84, 740-746.

Ghosh, S.S., Gehr, T.W.B., Ghosh, S., 2014. Curcumin and chronic kidney disease (CKD): Major mode of action through stimulating endogenous intestinal alkaline phosphatase. Molecules. 19, 20139-20156.

Kaushik, J., Tandon, S., Bhardwaj, R., Kaur, T., Singla, S.K., Kumar, J., Tandon, C., 2019. Delving into the Antiurolithiatic Potential of Tribulus terrestris Extract Through –In Vivo Efficacy and Preclinical Safety Investigations in Wistar Rats. Sci. Rep. 9, 1-3.

Lakhera, A., Ganeshpurkar, A., Bansal, D., Dubey, N., 2015. Chemopreventive role of Coriandrum sativum against gentamicin-induced renal histopathological damage in rats. Interdiscip. Toxicol. 8, 99-102.

Mishra, S., Aeri, V., Gaur, P.K., Jachak, S.M., 2014. Phytochemical, therapeutic, and ethnopharmacological overview for a traditionally important herb: Boerhavia diffusa linn. Biomed Res. Int. 2014, 808302.

Naggayi, M., Mukiibi, N., Iliya, E., 2015. The protective effects of aqueous extract of carica papaya seeds in paracetamol induced nephrotoxicity in male wistar rats. Afr. Health Sci. 15, 598-605.

Saleem, M., Javed, F., Asif, M., Baig, M.K., Arif, M., 2019. HPLC analysis and in vivo renoprotective evaluation of hydroalcoholic extract of cucumis melo seeds in gentamicin-induced renal damage. Med. 55, 107.

Sandeep, D., Krishnan Nair, C.K., 2010. Amelioration of cisplatin-induced nephrotoxicity by extracts of Hemidesmus indicus and Acorus calamus. Pharm. Biol. 48, 290-295.

Sharma, S., Baboota, S., Amin, S., Mir, S.R., 2020. Ameliorative effect of a standardized polyherbal combination in methotrexate-induced nephrotoxicity in the rat. Pharm. Biol. 58, 184-199.

Singh, B., Singh, Bikarma, Kishor, A., Singh, S., Bhat, M.N., Surmal, O., Musarella, C.M., 2020. Exploring plant-based ethnomedicine and quantitative ethnopharmacology: Medicinal plants utilized by the population of Jasrota Hill in Western Himalaya. Sustain. 12, 7526.

Singh, D., Chaudhuri, P.K., 2017. Chemistry and pharmacology of Tinospora cordifolia, in: Natural Product Communications. 12, 1934578X1701200240.

Table S2: Potential genes showing interaction with metabolites of NEERI KFT.

**Sr. no.**	**gene name**	**protein name**	**uniport id**
1.	Casp7	Caspase-7	P55210
2.	CASP3	Caspase-3	P42574
3.	BCL2	Bcl-2-related protein A1	Q16548
4.	BAAT	Bile acid-CoA:amino acid N-acyltransferase	Q14032
5.	CDH11	Cadherin-11	P55287
6.	UGT1A7	UDP-glucuronosyltransferase 1A7	Q9HAW7
7.	TP53	Cellular tumor antigen p53	P04637
8.	CHUK	Inhibitor of nuclear factor kappa-B kinase subunit alpha	O15111
9.	MAPK3	Mitogen-activated protein kinase 3	P27361
10.	UGT1A3	UDP-glucuronosyltransferase 1A3	P35503
11.	MMP2	72 kDa type IV collagenase	P08253
12.	GATA3	Trans-acting T-cell-specific transcription factor GATA-3	P23771

13.	MAPK1	Mitogen-activated protein kinase 1	P28482
14.	MMP3	Stromelysin-1	P08254
15.	HNF4A	Hepatocyte nuclear factor 4-alpha	P41235
16.	MMP9	Matrix metalloproteinase-9	P14780
17.	SOD1	Superoxide dismutase [Cu-Zn]	P00441
18.	AGTR1	Type-1 angiotensin II receptor	P30556
19.	ACE2	Angiotensin-converting enzyme 2	Q9BYF1
20.	PRKCA	Protein kinase C alpha type	P17252
21.	IL6	Interleukin-6 receptor subunit alpha	P08887
22.	JUN	Transcription factor Jun	P05412

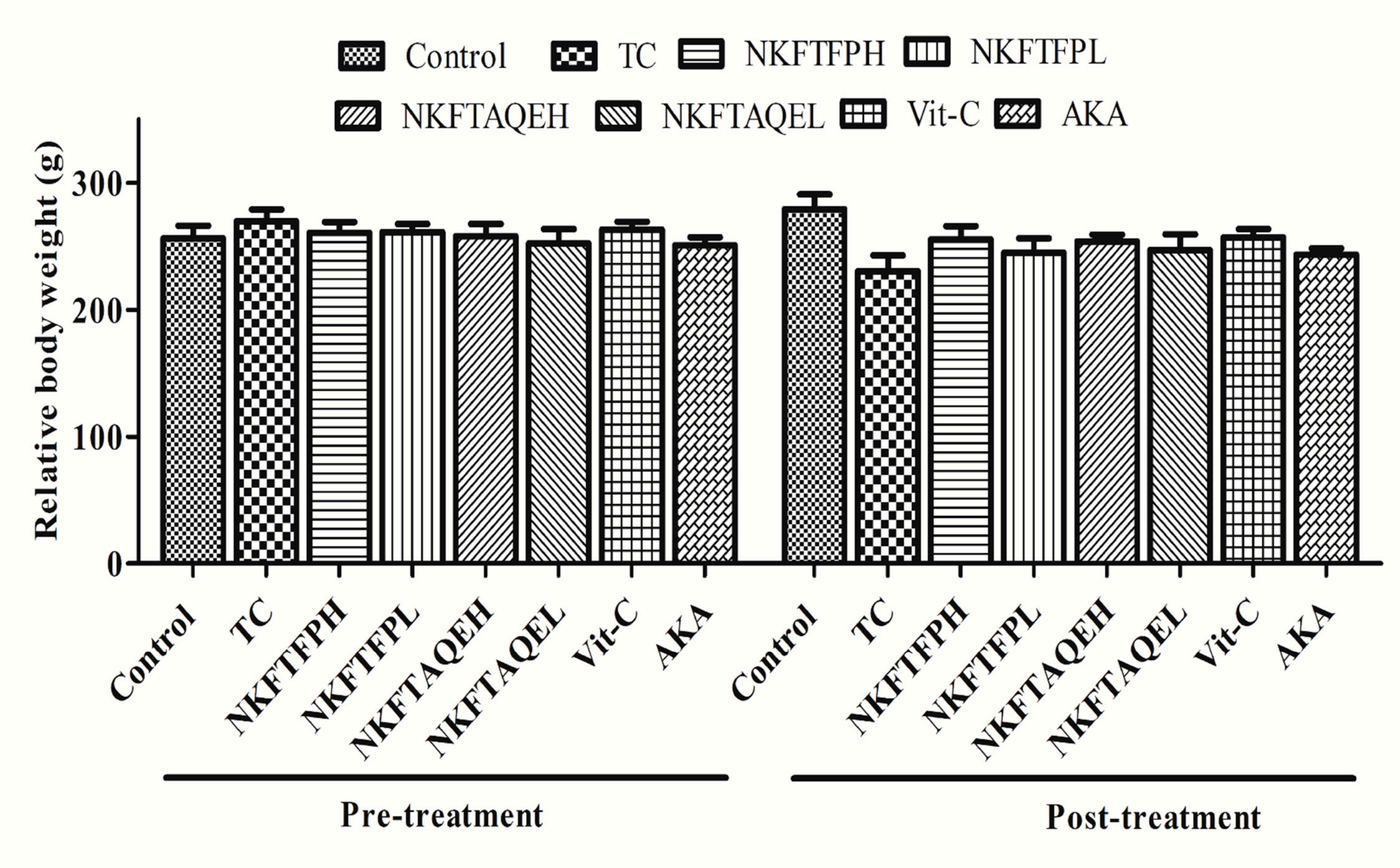

Figure S1: Assessment of pre-treatment and post-treatment relative body weight. The statistical representation were made as Mean ± SD (n=6) using One-way ANOVA followed by Tukey test. The comparisons were made to control and toxic, toxic to drug-treated groups. The significance level was observed at $p < 0.05$.
